# Politics of reproduction: a view from Israel on the Dobbs decision

**DOI:** 10.1186/s13584-022-00550-9

**Published:** 2023-01-24

**Authors:** Carmel Shalev

**Affiliations:** University of Haifa (Adjunct, Emeritus), Haifa, Israel

**Keywords:** Abortion, Autonomy, Choice, Dignity, Reproductive health, Medicalization, Repro-genetics, Jewish law, Patriarchy, Human trafficking, Women, Privacy, Liberty, Responsibility, Care

## Abstract

**Background:**

This opinion piece looks at the recent decision of the United States Supreme Court in Dobbs v. Jackson Women's Health Organization and then compares the law on abortion in the USA to the law in Israel on reproductive medicine in general.

The Dobbs decision validated a Mississippi state law that restricted access to abortion, while overruling the landmark precedent of Roe v. Wade on women's constitutional right to safe abortion. It declared that the US constitution does not confer upon women any right to abortion, whether pre- or post-viability, sending shockwaves throughout the world. It also had an immediate effect on women's reproductive health in the US.

**Main body:**

Women's right to reproductive freedom and to make decisions about their lives and their bodies is key to their hard-won equality. Still, abortion remains in ongoing controversy worldwide with legal barriers that impact upon the most vulnerable. In Israel, abortion is relatively available, accessible, affordable, and acceptable, in both law and practice. This is because of the lenient and nuanced stance of rabbinical authorities in the Jewish law tradition. This stance, together with Israel's post-Holocaust biblical culture of "be fruitful and multiply", also underlies its high rates of medically assisted reproduction for the treatment of infertility, including preimplantation genetic diagnosis of fertilized eggs. Women's bodies mediate all these repro-genetic technologies, in most cases for the benefit of others, not because of their own health needs. There is also concern about global practices and market forces that objectify women's bodies, exploit women and are harmful to their health, wellbeing, and dignity, carrying on outdated patriarchal patterns.

**Conclusion:**

Reproductive health policy ought to be based on an ethic of care and responsibility first and foremost for the women, as well as the children they choose to bring to life, in the spirit of the Jewish tradition that her life is of greater value than the fetus'. Women deserve control of their bodies and their lives and respect for the choices they make to the best of their judgment, which when it comes to abortion are mostly hard ones. They have a right to reproductive choice, freedom, autonomy, and dignity.

The views expressed in this perspective are those of the author.

## Background

The decision of the United States (US) Supreme Court in Dobbs v. Jackson Women's Health Organization (2022), to uphold a Mississippi law banning all abortions after 15 weeks gestational age except in life threatening medical emergencies and in the case of severe fetal abnormality,^1^ sent shockwaves throughout the world. In doing so the Dobbs court overturned the landmark ruling of Roe v. Wade (1973)^2^ on women's constitutional right to abortion within a protected zone of "privacy" that had been *stare decisis*, a binding precedent, for almost fifty years. It also overruled a subsequent decision of the US Supreme Court in Planned Parenthood v. Casey (1992) which ruled that a state law requiring that the spouse of a woman be notified if she seeks to terminate a pregnancy, was unconstitutional, while upholding women's constitutional right to safe abortion as a matter of personal liberty – the freedom to make "intimate choices" that are "central to personal dignity and autonomy".

In so doing the Dobbs court decided that the US constitution does not confer upon women any right to abortion at any stage of pregnancy, pre- or post-viability. It reasoned that the constitution makes no reference to abortion and stated that no right to abortion at any stage of pregnancy is implicitly protected by any constitutional provision. In other words, states might ban abortion from the moment of conception in the interest of "protecting the life of the unborn".

But the constitutional right to privacy is key to a patient's right to autonomy in making all medical choices and decisions, not just abortion.^3^ The American Medical Association condemned the Dobbs decision as “an egregious allowance of government intrusion into the medical examination room, a direct attack on the practice of medicine and the patient-physician relationship, and a brazen violation of patients’ rights to evidence-based reproductive health services.”^4^

## Main text

### Women's right to safe abortion

Roe v. Wade was delivered at a time that saw the decriminalization of abortion in many countries, and its reasoning was an inspiration for women all over the world to claim the right to be let alone in choosing whether or not and when to become a mother, and to have access to medical assistance and care in case of an unwanted pregnancy. The Roe court found that the constitutional right to privacy in matters of marriage, family and reproduction covered a woman's decision to terminate a pregnancy, and that this decision was inherently and primarily a medical one. In overturning Roe and ruling there was no constitutional protection of a woman's right to safe abortion, the Dobbs court provided a prime example of judicial activism. It went far beyond what was necessary to rule on the strict facts of the case before it, which concerned a specific law in the state of Mississippi that prohibited second trimester abortion, after 15 weeks of pregnancy, in the interests of the "unborn human being".

Roe had laid out a trimester scheme under which a woman had a right in the first trimester to decide to terminate a pregnancy, solely at her discretion; in the second trimester, protection of her health interests could justify medical regulation of abortion but not its prohibition; and only in the third trimester, post-viability, was there a legitimate state interest in protecting "potential life", (even though outside abortion law the unborn has never been treated as a legal person or holder of rights). In the last three months of pregnancy, therefore, states might outlaw abortion except when necessary to preserve the health of the mother as well as her life. In other words, even after viability the health and life of the pregnant woman must always come before any interest in the life of the fetus.

The Dobbs court came and declared that Roe's "critical distinction … between pre- and post-viability" was unjustified and "arbitrary". It also narrowed the causes for which late-term abortion might be approved to cases of actual threat to the mother's life rather than her general health, "protecting the life of the unborn" was more important. Overnight restrictive abortion laws in nine states came into effect. One month later a ten-year-old girl, a child who lived in a state where abortion was now illegal and who became pregnant from being raped by a grown man, had to cross the border to a neighboring state to gain access to the medical procedure and health care she needed.^5^

Roe did not invent the constitutional right to reproductive privacy. An earlier judgment, Griswold (1965), had recognized a constitutional zone of marital privacy in striking down a state law that prohibited the use of contraceptives by married couples.^6^ And Eisenstadt (1972) had found unconstitutional a law that prohibited the prescription and distribution of contraceptives other than to married couples as a violation of the right to privacy. "If the right of privacy means anything," the court said there, "it is the right of the *individual*, married or single, to be free from unwarranted governmental intrusion into matters so fundamentally affecting a person as the decision whether to bear or beget a child."^7^

But over the years since Roe, there had been ongoing attempts in the US to enact anti-abortion laws at the state level, together with a flow of cases challenging their constitutionality. This was an expression of the politics of abortion in the US, the pro-choice/pro-life social divide, and the trend towards ultra-right fundamentalism, which led among other things to the political appointment of the conservative judges who delivered the Dobbs decision.

The overall impact on women's health in the USA is a matter of concern, especially for those living in poverty who lack resources to seek out-of-state abortion and are more likely to resort to unsafe abortion services. Also, the health of women with chronic illnesses and disabilities might be compromised since they are at high risk for pregnancy complications that do not amount to life threatening emergencies. And there is concern about a chilling effect on the medical care women receive in cases of incomplete miscarriages of a wanted pregnancy, which are treated with the very same procedures used for abortion.^8^

### Reproductive freedom and equality

The right of women to make autonomous decisions about their lives is a relatively new development in law. Until around one hundred and fifty years ago women were regarded as chattels, the property of their fathers or husbands, not as legal persons in their own right. That was the law of patriarchy which reigned since the beginning of written history. Women's role was to serve as a vessel for men's sexual needs and reproduction, and the institution of marriage was a means of controlling women in order to overcome the uncertainty of biological fatherhood. In biblical law for instance a married woman who committed adultery was forbidden to both her lover and her husband and her child branded as bastard, while adulterous married men did not even bear legal responsibility for their illegitimate children. And only at the end of the twentieth century did the law acknowledge that a married man might be held criminally responsible for assaulting or raping his wife.

Women's right to control their lives and their bodies is the essence of their hard-won equality. The basis for the Roe ruling on women's right to privacy in abortion was the 14th amendment to the US constitution which was adopted towards the end of the nineteenth century to abolish slavery and its practices of breeding children for sale. Slaves became free to decide for themselves whom to marry and parent children with, even if this was the right of men and it would take many more years until women in general gained political and legal rights to equality as autonomous subjects.

But despite women's gradual emancipation and liberation over the course of the twentieth century, the issue of abortion remains controversial and sensitive around the world. Thus, for example, the Convention on the Elimination of All Forms of Discrimination against Women (CEDAW) does not mention abortion in so many words, although it recognizes the rights of women "to decide freely and responsibly on the number and spacing of their children and to have access to the information, education and means to enable them to exercise these rights."^9^

Likewise, the CEDAW committee's 1999 general recommendation on women's right to health^10^ referred only vaguely to the decriminalization of abortion, stating that it is discriminatory "to refuse to provide legally for the performance of certain reproductive health services for women" (s. 11), while "laws that criminalize medical procedures only needed by women" are barriers to their right to access appropriate health services (s. 14). At the same time, requirements for preliminary spousal authorization before abortion, like any other medical procedure or health care service, are a discriminatory barrier in access to services (s. 21). And acceptable services are those that are delivered in a way that ensures that a woman gives her fully informed consent, respects her dignity, guarantees her confidentiality and is sensitive to her needs and perspectives (s. 22).

Legal barriers to abortion impact the most vulnerable women, the least educated, those living in poverty in countries with limited access to contraception and high rates of maternal mortality and morbidity. These women will often resort to unsafe abortion, a leading and preventable cause of maternal deaths and health risks. According to the WHO, over half of all pregnancies worldwide are unintended, and around 45% of all abortions are unsafe, of which 97% occur in developing countries and low resource settings.^11^ Imagine a young woman, a teenager, pregnant from incest or rape, or having been forced into an underage marriage, without the wherewithal to do anything about it. If women have a right to safe motherhood, they need access to reproductive health services that include contraception, safe abortion services and post-abortion care, in addition to care throughout pregnancy, birth and its aftermath, if that is their choice.

### Abortion in Israel

When Israel was established in 1948 it inherited British Mandate law under which abortion was a criminal offence. But already in the early 1950s the then-attorney-general, Haim Cohn (who was to become a supreme court justice known for his liberal views), adopted a policy to not prosecute the crime, and in practice it was available in hospitals with the approval of a medical committee as well as in private clinics, as it is today. The law itself was eventually amended in 1977 to allow abortion on cause in a variety of circumstances, when approved by a committee of two doctors and a social worker.^12^

Therapeutic indications for legal abortion refer to the woman's physical or emotional health and wellbeing and to the risk of fetal anomalies. There are also social indications, such as age (under 17 and over 40) and being the victim of rape or incest, or unmarried. The need for committee approval is a bureaucratic hurdle, and abortion remains illegal in principle for married women, but in 2020, for example, the committees approved 99.6% of the applications they received.^13^ And shortly after the Dobbs decision, Israel's parliament backed a ministry of health proposal to ease abortion policy before the 12th week of pregnancy and allow women access to abortion medication (the day-after-pill) from community health providers, with public funding and without need for committee approval.^14^

At the same time abortion rates in Israel are relatively low compared to other countries and on a continuous decline, while fertility rates are relatively high. It has a popular culture of "be fruitful and multiply and fill the land" according to God's commandment after creating man, male and female, in his own image,^15^ and in the shadow of the Holocaust and its six million victims. Israel is also a world leader in bio-tech medicine including innovative repro-genetic technologies, with generous national health insurance coverage for almost unlimited access to medically assisted reproduction, including in vitro fertilization (IVF) and related interventions. A major reason for the cultural acceptance of abortion in Israel is the view of halakha, Jewish law. While ultra-orthodox rabbis take a strict stance on end-of-life medical care, which led to legal constraints on dying patients' medical choices because of the paramount value of the sanctity of life, the traditional rabbinical view of the beginning-of-life and the moral status of the embryo and fetus is nuanced and lenient.

Under Jewish law, according to the Talmud, life begins at forty days and until then the fetus is no more than "water in the world".^16^ If that is the case, there is no problem with so-called test-tube babies, nor with early termination of a pregnancy. This is strikingly different from the Catholic view that life begins at the very moment of conception, so that a fertilized egg has a soul and therefore IVF is frowned upon as sinful since it produces surplus embryos, not to mention its use for preimplantation genetic diagnosis.

Even in late-term pregnancy according to halakha, the woman's health and needs are of the utmost concern and her life is of more value than the fetus'. This is the classic interpretation by the medieval commentator Rashi of a biblical verse that refers to a "disaster" when a pregnant woman is hurt or dies in a brawl between her husband and another man. If she dies the husband can claim damages according to her market value and with added value because she was with child. But if the harm caused is that she miscarries and loses the fetus, he has no right to damages for the loss of its life.^17^ Moreover, in the explicit context of abortion the mother's life takes priority over the fetus'. Even when the woman is in actual labor the mother's life should be saved at its expense, says the Mishnah, a third century codification of oral Jewish law. The image is graphic: "If a woman is in trouble giving birth, they cut up the child in her womb and remove it limb by limb, because her life comes before its life."^18^

Over the decades since Israel's abortion law was amended in 1977 only one case came before its supreme court in 1981, Anonymous v. Anonymous.^19^ The issue was spousal consent. The wife there applied for an abortion which the committee approved, but the husband claimed standing before the committee. At his request a district court issued a temporary injunction against performing the abortion and ordered the committee to grant him a hearing and review its decision. The wife appealed to the supreme court which ruled by majority opinion that the husband has no standing in the abortion decision, certainly no right of veto, given the overriding interests of the woman. Therefore, he had no right to be heard by the committee, although it still might grant him a hearing, at its discretion.

Other jurisdictions had reached similar conclusions. A court in England decided in Paton (1978) that the father did not have any right to be consulted under the statute that reformed abortion law there in 1967.^20^ And in the US, the supreme court in Danforth (1976) had ruled that the requirement of spousal consent in writing for first-term abortion was unconstitutional.^21^ However, as mentioned, the Dobbs court reversed the 1992 Casey decision that had found a requirement of spousal notification (as opposed to consent) to be unconstitutional.

### The medicalization of reproduction

Reproductive relations are structured by deep-set patriarchal attitudes that allowed men to control women as their property rather than respect and treat them as equals. Women's capacity to conceive, bear and bring children into the world and then raise them was a key but depreciated resource. Women's labor is still undervalued—to this day their unpaid reproductive, parenting, and domestic work is not accounted for in the economic calculus of gross national product. But reproductive relations have also undergone radical transformation over the last century and a half with the medicalization of reproduction.

Abortion was criminalized in the US in the second half of the nineteenth century as a political move by an emerging medical profession that wished to claim moral authority over matters of life and death and exclude competing health practitioners, including the midwives who had until then taken care of their sisters in labor. To this day in Israel, birthing in hospital is the norm while births with midwives at home or at birth centers face legal and professional hurdles. In 2019, its national bioethics council recommended that these hurdles be removed and replaced with a midwifery paradigm of care,^22^ but its recommendation remains a dead letter, yet to be taken up.^23^

Since the second half of the twentieth century, medical technology has become ever more sophisticated and available in ways that affect numerous personal choices about life and death, including choices about bringing a child into the world. Medical contraception allowed sex without reproduction. Artificial insemination – the forerunner of IVF—allowed reproduction without sex as well as sperm donation for treatment of male infertility, while intruding into the privacy of marital relations. This and further technological developments produced a new body of reproductive law. For example, third-party reproductive collaborations—sperm donation, surrogate mother arrangements and egg donation – are supported by a distinction between biological and legal parenthood. Similarly, the standard of fetal viability for legal abortion is reliant on neonatal medical technology.

The discovery of IVF was a major medical innovation for which Robert Edwards was awarded the 2010 Nobel prize. The procedure was first developed to treat women who could not get pregnant because their fallopian tubes were blocked. The solution was to extract eggs from the patient's ovaries, fertilize them in vitro, and return the fertilized egg to her womb. The first IVF child was born in England in 1978, in Israel it was in 1982. It soon became a standard procedure and a platform for multiple uses including treatment of male infertility (with intracytoplasmic sperm injection (ICSI) replacing artificial insemination with donor sperm), third-party reproductive collaborations, preimplantation genetic diagnosis of embryos, and stem cell research. IVF was also the platform for cloning, which the international community considered incompatible with human dignity and called to prohibit in the United Nations Declaration on Human Cloning (2005),^24^ referring to the Universal Declaration on the Human Genome and Human Rights (1997).^25^ Israel also has a law that bans cloning, at least for the time being – it is a provisional prohibition that is extended every five years.^26^

Meanwhile, since the early 1990s, the number of IVF interventions in Israel has been on an upward trend. In 2018, according to the Ministry of Health,^27^ women in Israel underwent over 48 thousand treatment cycles (almost three times more than the applications for induced abortion in the same year)^28^ which resulted in over nine thousand newborns. Success rates remain steady at around one out of five treatment cycles resulting in live births, with some women undergoing unlimited rounds of publicly funded treatment. Women's bodies are necessary to mediate these procedures which are not trivial, and in most cases nowadays they are not the direct beneficiaries of the interventions but undergo them for the benefit of others, whether ICSI for an infertile partner or for the purpose of egg donation or surrogacy, all of which might take a toll on women's physical and emotional health, time will tell.

Proliferation of the technology has been driven in large part by private profit-seeking markets, increasingly global, rather than being guided by public policy and concern for women's dignity, health, and wellbeing. There are concerns about the commodification of embryos and newborns with technologies that allow genetic selection and modification, and fear of a market eugenics driven by the selfish greed of entrepreneurs and whim of consumers. Thousand-year-old patriarchal habits of mind die hard within the context of market conditions that are ripe for the exploitation and objectification of women, especially in the poor economies of under-developed countries. Global practices include transnational surrogacy which easily amounts to trafficking in women and has been banned for that reason in countries like India and Nepal. Gay couples were major clients of this business in Israel, until it amended its law on surrogacy to allow them to make such agreements with local women.

## Conclusions

Tampering with human reproduction even with the best of intentions raises a plethora of ethical and moral questions around reproductive relations, and challenges long-standing religious and moral sensibilities about the meaning of human nature and forms of human being.^29^ Yet despite the impressive achievements of reproductive medicine, women continue to be those who carry pregnancies and give birth to newborns. They are also, in the main, those who care for them through childhood. Their wellbeing should be paramount.

Individual liberty and rights are essential, precious, and invaluable, but they are based on self-interest and their language pits the woman against the fetus. What we need is an ethic of care and responsibility for the women, as well as the children they choose to conceive, bear and birth; an ethic of care and responsibility that comes from empathy and aims to minimize harm, especially for the most vulnerable. We need a policy that aims primarily to protect the rights of women to reproductive health, in the spirit of the Jewish tradition that her life is of greater value than the fetus'.

The change in social norms of gender equality over the twentieth century, and the notions that women are persons who hold equal rights to freedom and dignity, who can exercise moral agency and make autonomous decisions about bringing a child into the world, are revolutionary. This notion of moral agency within an ethic of care and responsibility was the key theme of my book, Birth Power—The Case for Surrogacy.^30^ Women know well what it means to care and be responsible for another. They deserve control of their bodies and their lives and respect for the choices they make to the best of their judgment, which when it comes to abortion are mostly hard ones. They deserve our care and responsibility. The Dobbs decision reminds us that this is not to be taken for granted, in face of fundamentalist religious political trends which find expression in different forms of prejudice and discrimination, including in Israel.

## Data Availability

Not applicable.

## Abbreviations

CEDAW: Convention on the Elimination of all forms of Discrimination Against Women
ICSI: Intracytoplasmic sperm injection
IVF: In vitro fertilization
US: United Sates of America

## Footnotes

1 Dobbs v. Jackson Women's Health Organization https://www.supremecourt.gov/opinions/21pdf/19-1392_6j37.pdf

2 Roe v. Wade 410 US 113 (1973).

3 Quinlan (1976) and Cruzan (1990) also relied on the right to privacy as regards rights of patients to refuse life-saving interventions, including through advance directives.

4 https://www.ama-assn.org/about/leadership/dobbs-ruling-assault-reproductive-health-safe-medical-practice

5 New York Times, 14 July 2022. https://www.nytimes.com/2022/07/14/business/media/10-year-old-girl-ohio-rape.html

6 Griswold v. Connecticut 381 US 479 (1965).

7 Eisenstadt v. Baird 405 US 438 (1972).

8 Bonnie Steinbock, Abortion, Hastings Center (https://www.thehastingscenter.org/briefingbook/abortion/) briefing.

9 Article 16 (e) of the Convention on the Elimination of all forms of Discrimination Against Women (CEDAW) (1981). See https://www.ohchr.org/sites/default/files/Documents/ProfessionalInterest/cedaw.pdf.

10 CEDAW General Recommendation No. 24 on Women and Health, 20th session (1999). See https://tbinternet.ohchr.org/Treaties/CEDAW/Shared%20Documents/1_Global/INT_CEDAW_GEC_4738_E.pdf.

11 WHO fact sheet on Abortion, 25 November 2021. See https://www.who.int/news-room/fact-sheets/detail/abortion.

12 Criminal Law Amendment (Terminations of Pregnancy), 1977.

13 Israel Central Bureau of Statistics press release on Applications to Abortion Committees in 2020, 5 January 2022. See https://www.cbs.gov.il/he/mediarelease/pages/2022/%D7%A4%D7%A0%D7%99%D7%95%D7%AA-%D7%9C%D7%95%D7%95%D7%A2%D7%93%D7%95%D7%AA-%D7%9C%D7%94%D7%A4%D7%A1%D7%A7%D7%AA-%D7%94%D7%99%D7%A8%D7%99%D7%95%D7%9F-%D7%91%D7%A9%D7%A0%D7%AA-2020.aspx [in Hebrew].

14 Ido Efrati, Israel to ease restrictions on medical abortions before 12th week of pregnancy, Haaretz (https://www.haaretz.com/israel-news/2022-06-27/ty-article/.premium/israel-to-ease-restrictions-on-medical-abortions/00000181-a4dc-d0ff-a1f7-b5fc69c70000), June 27, 2022.

15 Genesis 1:28.

16 Talmud, Yevamot 89:2.

17 Exodus 21:22–25.

18 Mishnah, Ohalot 7.

19 Anonymous v. Anonymous (1981) 35 PD (3) 57.

20 Paton v. Trustees of BPAS [1978] 2 All ER 987.

21 Planned Parenthood of Central Missouri v. Danforth 428 US 52 (1976).

22 Freedom of Choice and Human Rights in Birthing, Israel National Bioethics Council, Position Paper #8 (2019). See https://www.health.gov.il/PublicationsFiles/Bioethics-human-rights.pdf [in Hebrew].

23 Prohibition of Genetic Intervention (Human Cloning and Genetic Alteration of Gametes) Law, 1999.

24 United Nations Declaration on Human Dignity, UN General Assembly Sixth Committee, 59th session, 16 February 2015.

25 Universal Declaration on the Human Genome and Human Rights, UNESCO General Conference, 29th session, 11 November 1997. See https://www.ohchr.org/en/instruments-mechanisms/instruments/universal-declaration-human-genome-and-human-rights.

26 Prohibition of Genetic Intervention (Human Cloning and Gamete Genetic Alteration) Law, 1999.

27 In-Vitro Fertilization Treatments 1990–2018, Israel Ministry of Health Information Unit. See https://www.gov.il/BlobFolder/news/11082020_02/en/NEWS_spokesmanship_ivf1990-2018.pdf [in Hebrew].

28 Israel Central Bureau of Statistics press release (18 December 2019). See https://www.cbs.gov.il/he/mediarelease/pages/2019/%D7%A4%D7%A0%D7%99%D7%95%D7%AA-%D7%9C%D7%95%D7%95%D7%A2%D7%93%D7%95%D7%AA-%D7%9C%D7%94%D7%A4%D7%A1%D7%A7%D7%AA-%D7%94%D7%99%D7%A8%D7%99%D7%95%D7%9F-%D7%91%D7%A9%D7%A0%D7%99%D7%9D-2018-2017.aspx [in Hebrew].

29 Carmel Shalev, Reproductive Ethics (2015), in ten Have (ed.) Encyclopedia of Global Bioethics (https://www.researchgate.net/publication/321716321_Reproductive_Ethics), Springer.

30 Carmel Shalev, Birth Power – The Case for Surrogacy, (Yale University Press, 1989).

